# Unsupervised machine learning identifies predictive progression markers of IPF

**DOI:** 10.1007/s00330-022-09101-x

**Published:** 2022-09-06

**Authors:** Jeanny Pan, Johannes Hofmanninger, Karl-Heinz Nenning, Florian Prayer, Sebastian Röhrich, Nicola Sverzellati, Venerino Poletti, Sara Tomassetti, Michael Weber, Helmut Prosch, Georg Langs

**Affiliations:** 1 Computational Imaging Research Lab, Department of Biomedical Imaging and Image-guided Therapy, Medical University of Vienna, Spitalgasse 23, 1090 Vienna, Austria; 2 Department of Biomedical Imaging and Image-guided Therapy, Medical University of Vienna, Währinger Gürtel 18-20, 1090 Vienna, Austria; 3 Unit “Scienze Radiologiche”, Department of Medicine and Surgery (DiMeC), University of Parma, Parma, Italy; 4 Department of Thoracic Diseases, Morgagni-Pierantoni Hospital, Forlì, Italy; 5 Department of Respiratory Diseases and Allergy, Aarhus University Hospital, Aarhus, Denmark

**Keywords:** Idiopathic pulmonary fibrosis, Unsupervised machine learning, Tomography, X-ray computed

## Abstract

**Objectives:**

To identify and evaluate predictive lung imaging markers and their pathways of change during progression of idiopathic pulmonary fibrosis (IPF) from sequential data of an IPF cohort. To test if these imaging markers predict outcome.

**Methods:**

We studied radiological disease progression in 76 patients with IPF, including overall 190 computed tomography (CT) examinations of the chest. An algorithm identified candidates for imaging patterns marking progression by computationally clustering visual CT features. A classification algorithm selected clusters associated with radiological disease progression by testing their value for recognizing the temporal sequence of examinations. This resulted in radiological disease progression signatures, and pathways of lung tissue change accompanying progression observed across the cohort. Finally, we tested if the dynamics of marker patterns predict outcome, and performed an external validation study on a cohort from a different center.

**Results:**

Progression marker patterns were identified and exhibited high stability in a repeatability experiment with 20 random sub-cohorts of the overall cohort. The 4 top-ranked progression markers were consistently selected as most informative for progression across all random sub-cohorts. After spatial image registration, local tracking of lung pattern transitions revealed a network of tissue transition pathways from healthy to a sequence of disease tissues. The progression markers were predictive for outcome, and the model achieved comparable results on a replication cohort.

**Conclusions:**

Unsupervised learning can identify radiological disease progression markers that predict outcome. Local tracking of pattern transitions reveals pathways of radiological disease progression from healthy lung tissue through a sequence of diseased tissue types.

**Key Points:**

• *Unsupervised learning can identify radiological disease progression markers that predict outcome in patients with idiopathic pulmonary fibrosis*.

• *Local tracking of pattern transitions reveals pathways of radiological disease progression from healthy lung tissue through a sequence of diseased tissue types*.

• *The progression markers achieved comparable results on a replication cohort*.

## Introduction

Idiopathic pulmonary fibrosis (IPF) is the most frequent type of idiopathic interstitial pneumonias (IIP) and accounts for a fifth of all cases of interstitial lung diseases (ILDs) [1, 2]. Although IPF has been considered rare, a review based on 34 studies of IPF incidence and mortality has shown an incidence rate from 0.2 to 93.7 per 100,000 persons per year [3, 4]. Studies have estimated that without treatment, the median survival among persons with IPF is 3–5 years after diagnosis [5]. Even though the disease cannot be reversed, the radiological disease progression can be slowed down with disease-modifying drugs such as pirfenidone and nintedanib [6]. CT plays a central role in the diagnosis of IPF; the official ATS/ERS/JRS/ALAT clinical practice guideline of IPF states that in case of a typical usual interstitial pneumonia (UIP) pattern on CT, a lung biopsy is considered unnecessary [7]. However, in many cases, there is still a considerable overlap of radiologic, clinical, and physiologic appearance with other ILDs, such as non-specific interstitial pneumonia and hypersensitivity pneumonitis [8, 9]. Consequently, there is substantial disagreement between radiologists, clinicians, and pathologists regarding final diagnosis of ILDs [10]. Most importantly, IPF and other ILDs have distinctly different prognoses and treatment options which emphasizes the importance of correct diagnosis [8].

Beside the difficulties in diagnosing IPF, the prediction of radiological disease progression is even more challenging as disease courses in IPF are quite divergent. Imaging features associated with a worse prognosis have been reported to be the extent of bronchiectasis, the extent of honeycombing and the volume of vessel-associated structures [11].

Given the inter-observer variability in the recognition of these features, the routine usability of these features is limited without the support of dedicated software [12]. Furthermore, most of the investigated imaging features observed hitherto are based on well-established CT patterns, which are not necessarily the most predictive features, either due to the lack of a link to biological radiological disease progression, or difficulty of identification and associated inter- and intra-reader variability [13].

Therefore, techniques for the identification of quantitative imaging markers of IPF radiological disease progression that predict future disease course and outcome are highly desirable.

It was the aim of this study to develop an unsupervised machine learning approach to identify novel radiological disease progression imaging marker patterns and evaluate if these patterns predict outcome.

## Material and methods

This retrospective study was approved by the Institutional Review Board of the Medical University of Vienna (Ethics Committee number 1463/2017). The local Institutional Review Board waived the informed consent.

### Study population

**Fig. 1:**
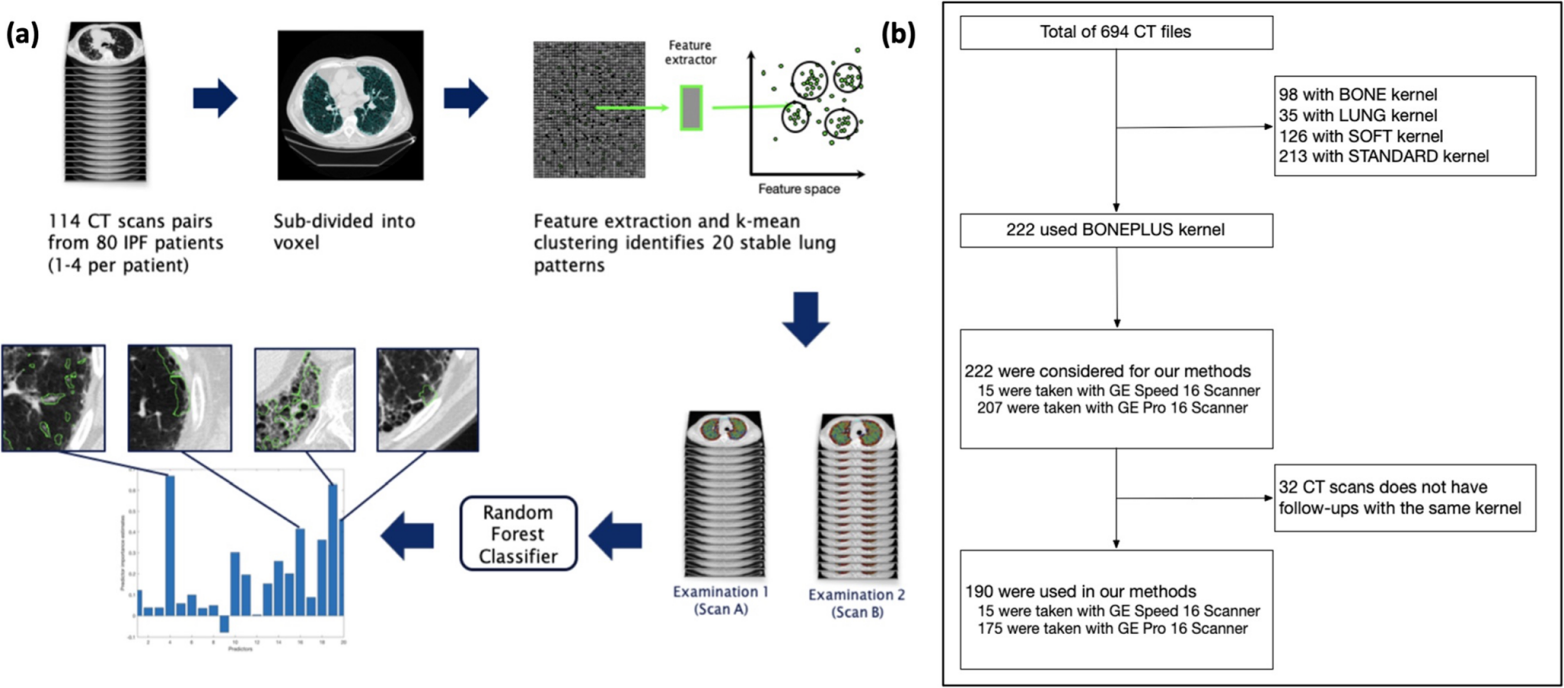
Overview of the algorithm and dataset. **a** First, unsupervised learning selects marker candidates, which results in most significant progression markers. **b** This flowchart represents the selection of enrolled scans

For the study cohort, IPF patients with diagnosis of IPF between December 2011 and October 2014 were retrospectively retrieved from the electronic registers of an Italian referral center (Ospedale Morgagni di Forlì, Italy, *n* = 76). Inclusion criteria were as follows: (1) availability of at least two consecutive HRCT examinations per patient performed at least 6 months interval; (2) usage of a high-frequency reconstruction kernel (BONEPLUS) with a slice thickness of ≤ 1.25 mm for both examinations. Following these inclusion criteria, a total of 76 patients (f/m: 19/57) were included, as only in these patients follow-up scans with the same reconstruction kernel were available (Fig. 1b). For a sub-cohort of 74 patients, survival data was available. The patient characteristics of the entire study cohort can be seen in Table 1.

**Table 1:** Patient characteristics of the entire study cohort

	Study cohort (*n* = 76)	Replication cohort (*n* = 18)	*p* value
Age at first CT, years			
Mean age	65.10 ± 8.73	67.67 ± 12.39	.305
Age range	34–82	29–83	..
Gender			
Female	19	8	.101
Male	57	10	.101
Lung function test			
FVC (mean ± sdt)	81.63 ± 18.96	77.64 ± 27.17	.465
DLCO (mean ± sdt)	54.27 ± 15.60	47.32 ± 16.75	.097
Unknown	3	4	..
CT pattern			
Definite UIP	32	7	.803
Probable UIP	30	10	.215
Indeterminate for UIP	5	1	.873
Inconsistent with UIP	9	0	.124
Smoking status			
Active smoker	1	2	.033
Former smoker	46	11	.960
Never smoker	25	4	.379
Unknown	4	1	.960
Survival outcome			
Alive	20	4	.719
Dead	54	14	.569
Unknown	2	0	.484

As a replication cohort, we collected a retrospective cohort from a different center and country (*n* = 18, Vienna General Hospital, Austria). Patients in this dataset were diagnosed with IPF between April 2007 and April 2017. The inclusion criteria were the same as the study referral center, but with a different CT reconstruction kernel (B60f, B70f, B70s, I70f, I80s) since scanners were from a different manufacturer.

For both cohorts, the CT diagnosis was established by two experienced radiologists. The diagnosis of the IPF was made by the multidisciplinary ILDs boards of both institutions.

### Imaging data collection and acquisition

The study cohort dataset (Italy) was acquired with 2 CT scanners, a LightSpeed Pro 16, and a BrightSpeed 16 (both GE Healthcare). The CT examinations were performed in supine position in sustained deep inspiration. In case of more than two CT examinations per patient, each pair of consecutive CT scans was included. Therefore, an individual patient could have 1–4 pairs of scans. For the replication cohort, data was acquired with a Siemens Sensation Cardiac 64 scanner in supine position with deep inspiration. Each patient had 2 scans, one at the time of the diagnosis and another one at the last seen examination.

### Segmentation and follow-up registration

Lungs were automatically segmented in the CT data in a two-step approach. First, a threshold-based algorithm was used [14], and morphological area opening removed small structures such as small bronchi and vessels. The background was suppressed, and airways were extracted by trachea localization. If the algorithm failed the volume-based assessment criterion, as in cases of substantial high-density areas and lung scarring, a multi-template atlas–based segmentation approach was used to correct the segmentation [15]. This segmentation approach automatically selected an optimal templated lung transformation (VISCERAL Anatomy 3 [16]) using normalized cross correlation criteria and performed a non-linear registration from this selected transformation to a predefined target atlas with Ezyes [17].

After lung mask segmentation, pairs of consecutive CT scans were registered using Advanced Normalization Tools (ANTs) [18] to establish correspondence of positions in the lung imaging data for subsequent examinations of the same patient. Registration of corresponding positions was necessary to track the change of lung pattern classes over time and measure local transitions between pattern classes during radiological disease progression.

To reduce computational complexity, instead of processing each individual voxel of the slices, we over-segmented the lung mask into small parcels of the size of 5 mm × 5 mm × 5 mm — so-called supervoxels — using MonoSLIC [19]. Those supervoxels were extracted through k-means clustering performed on the monogenic phase detecting the locally dominant structure of the CT voxel regardless of the contrast and brightness of the image, resulting in a total of 1,578,788 supervoxels covering the entire lung cohort (Fig. 1a).

### Extraction of CT features and radiological disease progression marker candidates

We identified distinct lung appearance patterns occurring across the entire study population by unsupervised machine learning on all imaging data using a *bag of visual words approach* [20]. Computed tomography imaging data was received in the form of DICOM files, with gray values representing Hounsfield units. The gray value range was transformed to 0 to 255 before extracting image features. We calculated various statistical properties of the orientation-independent gray-level co-occurrence matrices for each *supervoxel*, resulting in so-called Haralick textural features [21], a 65-component vector per supervoxel. To reduce this high dimensionality, we used principal component analysis (PCA) retaining 95% of the overall variance resulting in a 9 dimensional feature vector per supervoxel (Fig. 1a).

K-means clustering in this 9 dimensional feature space assigned each supervoxel to one lung appearance pattern corresponding to a cluster. The optimal number of clusters was determined by repeatability testing, using Jaccard score [22] resulting in *k* = 20 clusters as the optimal choice. Finally, each lung was represented by the volume fraction covered by each of the 20 appearance patterns, resulting in a vector of 20 components. This *pattern signature* captures the overall texture composition of the lung.

### Identifying marker patterns of radiological disease progression and pathways of local tissue transition

IPF is associated with pulmonary fibrosis, a type of terminal pathological change in the lung, caused by chronic repetitive alveolar injury and results in excessive synthesis of extracellular matrix and replacement of normal parenchyma. While some types of pulmonary fibrosis are reversible, IPF exhibits progressive and irreversible development [23, 24].

To identify pattern signature components associated with radiological disease progression, we analyzed available pairs of subsequent CT scans of the same patient (scan A and scan B) with known acquisition dates. We trained a random forest (RF) [25] classification model with 500 trees to predict the correct temporal sequence of two scans (A, B) based on the difference of their pattern signatures (prediction result: A acquired after B or B acquired after A, ground truth during training based on the acquisition dates in the DICOM header). We used Gini importance to rank features regarding their predictive power for correct sorting. Since IPF is irreversible, the lung scarring captured in CT scans either remains the same or worsens. Thus, we hypothesize that features enabling correct temporal sorting capture radiological disease progression.

The ground truth for the correct temporal sequence was read from the CT DICOM-header. We used RF Gini importance to score the contribution of pattern signature components to the correct sorting of scans, and hypothesize that components — each associated with a lung tissue type with specific appearance — with high score are strongly associated with radiological disease progression.

### Predicting outcome based on the dynamics of pattern signatures

We used only the top 4 scored components determined previously together with their change between a pair of follow-up scans to form the *radiological disease progression signature*. We clustered patients into two groups using k-means clustering of their progression signatures. For each patient cluster, we assessed survival in a Kaplan-Meier analysis.

### Exploratory analysis of pattern transition pathways

We analyzed if the transition of lung tissue from one to a different pattern follows one or more specific sequences during the course of the disease. We determined the image signature component at each lung position in one scan, and the component at the corresponding position in the subsequent scan for all 1,578,788 supervoxels and all scan pairs in the study. This yielded a transition probability network. It captures how likely it is to transition from one of the 20 tissue patterns to another during radiological disease progression.

### Evaluation

To validate the radiological disease progression model, we tested if the machine learning model could correctly determine the temporal sequence of pairs of subsequent CT examinations based solely on the image signatures extracted from each of the two CT volumes, resulting in the “sorting accuracy” of the RF model.

To evaluate if inaccuracies stem from a lack of visible radiological disease progression, or algorithmic limitations, we compared the sorting accuracy of the RF model with the sorting accuracy of two experts with 17 years (expert 1) and 15 years (expert 2) of experience in thoracic radiology. The radiologists were shown pairs of follow-up scans in random order blinded for examination dates. Algorithm sorting accuracy was evaluated in leave-one-patient-out cross-validation, by training the machine learning model, and identifying radiological disease progression markers on 76 patients of the dataset, and automatically sorting the remaining pair of scans from a patient of the dataset with the trained RF model.

To assess the stability of radiological disease progression marker patterns, we randomly picked 20 subsets of 95% (*n* = 72) of the patient’s scan pairs in each run, to train the machine learning model, and tested if the ranking of the top informative radiological disease progression markers remained the same. The top-ranked prototypes were assessed and evaluated as image patches (250 × 250 pixels) by an expert for their content.

To evaluate if the progression signatures predict outcome, we assessed the hazard ratio (HR) between the two patient clusters identified based on the progression signatures. In the study cohort, for 74 patients, survival data was available, and analysis was performed on those patients. In a replication experiment, we processed the external validation data (*n* = 18) using the same 4 components of the progression signature, and assigned each new patient to one of the two existing patient clusters identified in the study cohort. We evaluated replicability by Kaplan-Meier analysis, analogously to the study cohort.

## Results

### Radiological disease progression can be detected with accuracy comparable to human expert readers

The machine learning model correctly identified the temporal sequence of 95 out of 114 CT scan pairs (83%). In comparison, the expert accuracy was 83% and 67%, respectively. Comparison of the mistakes of the machine learning model and the expert readers showed that 9 out of 19 (47%) cases erroneously sorted by expert 1 (E1) were also erroneously sorted by expert 2 (E2) (Table 2). The comparison of the model with human experts shows that 7 out of 19 (37%) errors made by the machine learning model (ML) were also made by expert 1, while 12 out of 19 (63%) ML model errors were also made by expert 2. Six out of 114 pairs were evaluated incorrectly by all three (2 readers and ML). The percentage of correct answers is different (*p* = .008) between the readers (E1, E2, ML). We used the generalized estimation equations model to evaluate the misclassification between the two readers, E1 (83% correct) and E2 (67% correct; *p* = .008), which shows to be significantly different, but we found no significant difference between ML and either of the two experts (*p* = .496 and *p* = .156).

**Table 2:** Comparison of errors of machine learning models with expert readers

	Reader 1	Reader 2	Overlap errors
R1 = expert 1, R2 = expert 2	19	38	9 (47.3% of R1, 23.6% of R2)
R1 = expert 1, R2 =ML	19	19	7 (36.8% of R1)
R1 = expert 2, R2 = ML	38	19	12 (31.57% of R1)

### Radiological disease progression markers are stable

**Fig. 2:**
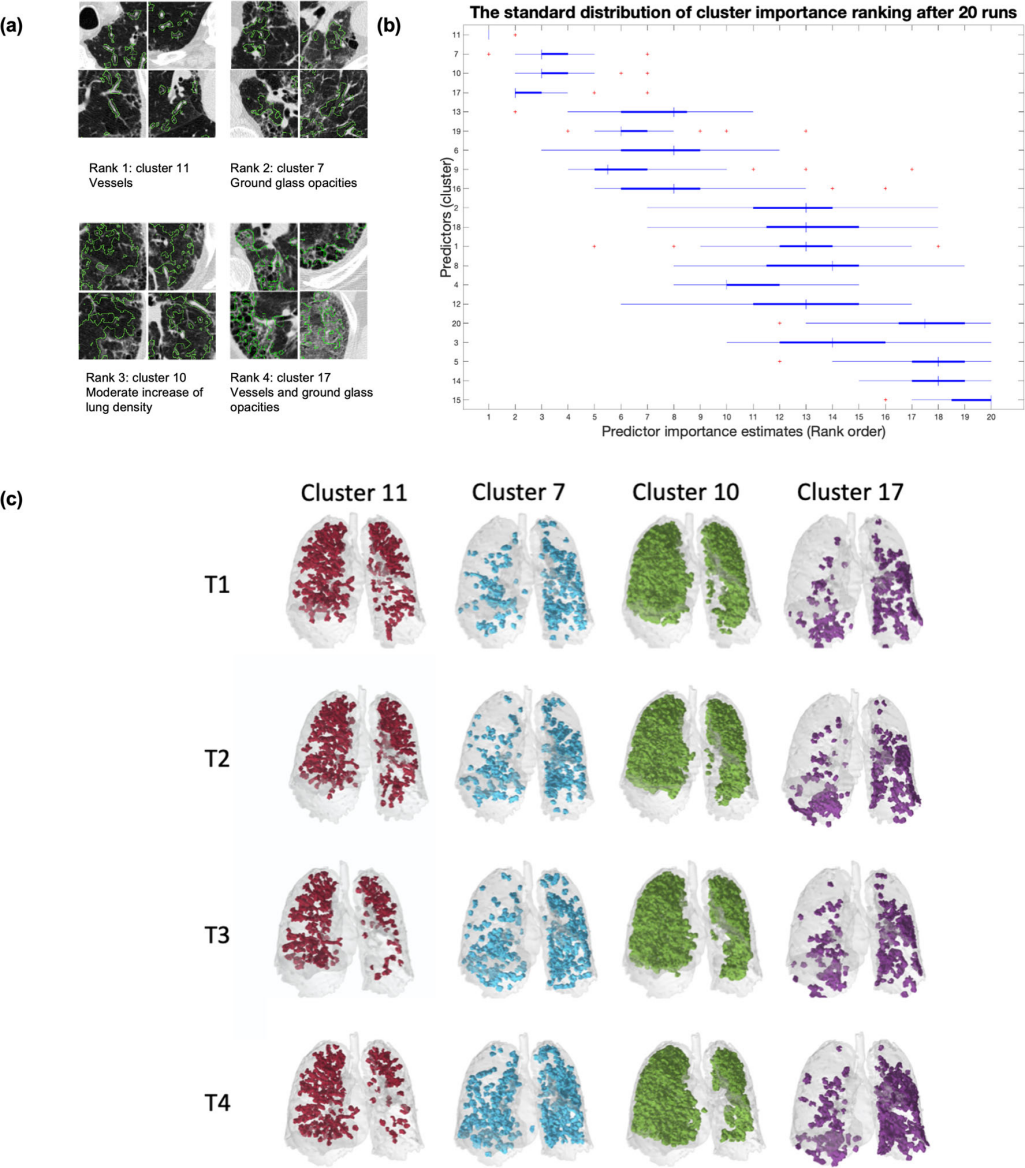
Evaluation of the stability of the progression markers. **a** The pattern example among the top 4 ranked pattern. **b** Most informative progression markers identified by the model, and the repeatability of this ranking after 20 runs of random 95–5% patient splits. The top 4 ranked patterns are stable across all runs. The ranking of less informative patterns fluctuates across runs. **c** The top 4 ranked cluster volume representation from a patient at 4 different time points

Figure 2 shows the rankings of the most informative radiological disease progression marker candidates identified by Gini importance [26] across 20 runs on randomized subsets. The top 4 prototypes were consistently top-ranked across all runs, containing vessels, ground glass opacities and increased density regions. Figure 2b depicts the average rank and rank standard deviation of all the prototypes sorted following their average ranking. Figure 2c illustrates the top 4 ranked cluster’s volume representations from a patient at 4 different time points. Example patches of those four patterns (11 - 7 - 10 - 17) from the same patient are shown in Fig. 2a.

### Progression signatures predict outcome

**Fig. 3:**
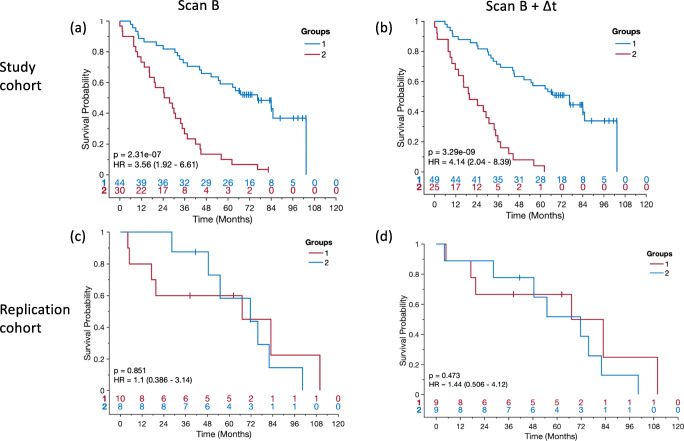
The survival study of Kaplan-Meier (KM) estimation of the most informative progression markers. **a** The KM curve based on markers of the scan B on the study cohort. **b** The KM curve based on markers of the scan B and the difference of the scan A and B on the study cohort. **c** The KM curve based on markers of the scan B on the replication cohort. **d** The KM curve based on markers of the scan B and the difference of the scan A and B on the replication cohort

Clustering patients based solely on their radiological disease progression signature results in two patient groups with markedly different outcomes (Figure 3). In the study cohort, clustering with the 4 static components of the signature leads to a HR = 3.56 (*p* < .01). Including the dynamic components (the difference between scans) results in higher HR = 4.14 (*p* < .01) between the two groups. When using the same progression signatures, and clusters, to process patients in the external validation cohort, the 4 static components and the full progression signature yield HR = 1.10 and HR = 1.44 (same trend as in study cohort, but not significant), respectively (Fig. 3). For the external validation, no re-training of patterns or clusters was performed.

### Transition pathways of local lung imaging patterns emerge during radiological disease progression

**Fig. 4:**
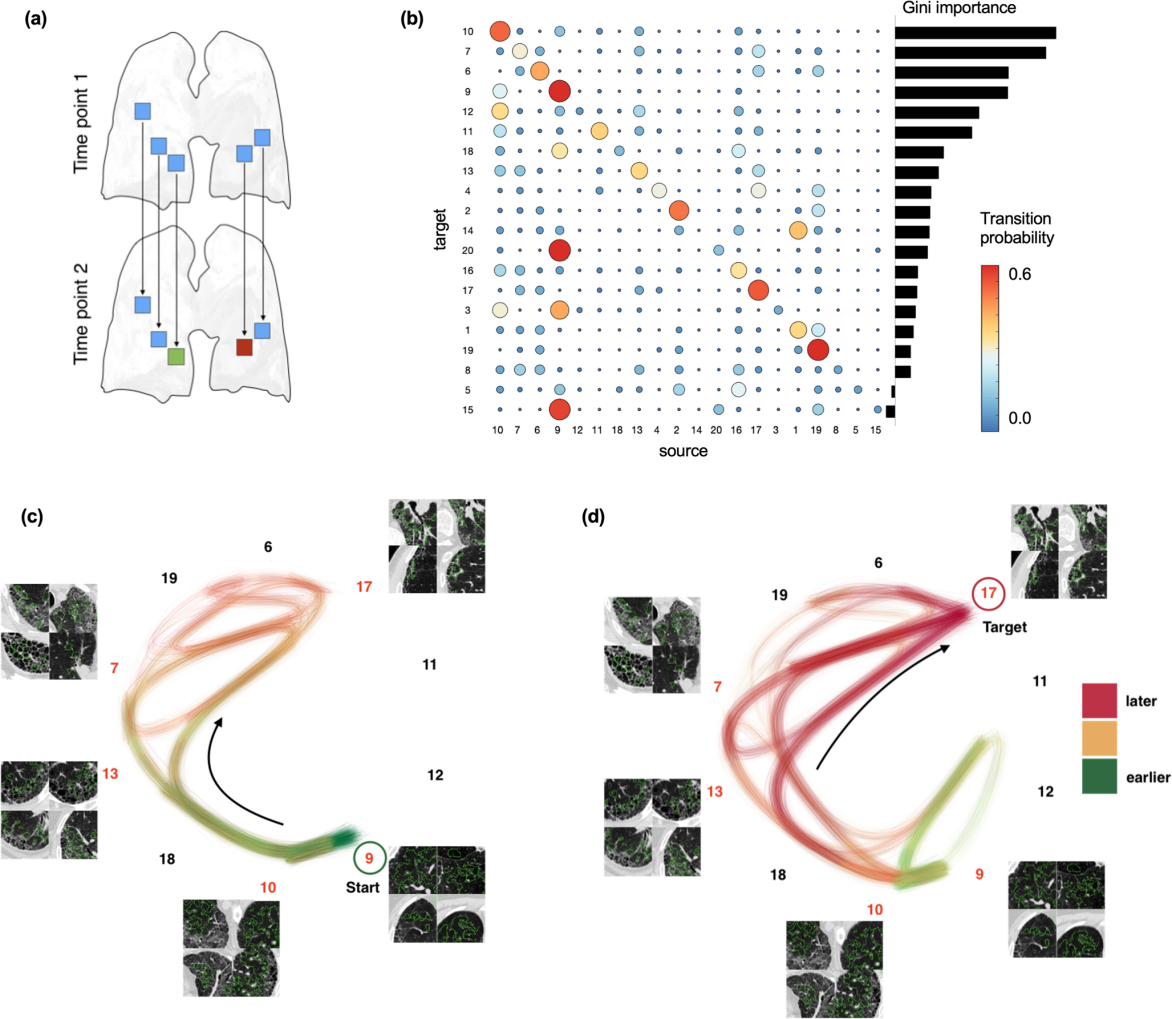
Pattern transition networks: mapping local pattern transition networks to reconstruct pathway candidates. **a** From the population of spatially matched follow-up pairs of lungs, we can observe local change of lung tissue from one to another pattern. (**b**) This enables obtaining a network of transition probabilities of lung patterns changing to others from one to the next examination time point. The matrix shows how likely a source pattern transitions to a target pattern. Red indicates high probability, blue low probability. These probabilities are generated by an underlying latent transition network that exhibits transition pathways shown in this figure. For the top ranked most informative patterns, we plot two pathways to illustrate this model. **c** Pathways originating from a healthy pattern (cluster 9), and (**d**) pathways ending in vessels and ground glass pattern (cluster 17). Arrows point at dominant directions in the graph

Exploratory analysis of progression pathways revealed a network of transition probabilities. They quantify how likely lung tissue is changing from one pattern to another during radiological disease progression (Fig. 4a and b). The latent transition network revealed three types of patterns. Relatively *stable patterns*, such as 9, 10, and 19, remained primarily unchanged over time. *Volatile patterns*, such as 4, 5, or 8, changed to other patterns more frequently, and *transient patterns* that transitioned to a specific different pattern more often than staying the same such as for instance 12, 14, 15, 18, or 20.

A simulation of particles entering a corresponding random walk results in possible pathways of disease pattern progression through the transition network. For illustration, we reconstructed two pathway visualizations. In Fig. 4c, we initiated particles in cluster 9 and observed how they transition the network among 10 patterns. The most dominant paths were 9 - 10 - 13 - 17, or 9 - 10 - 13 - 7, reflecting a transition from one to another healthy pattern towards minimal ground glass and reticular patterns and finally a pattern consisting of a mix of vessels and severe ground glass opacities (cluster 17 and cluster 7). A simulation in the reverse direction (Fig. 4d) asked for pathways ending in cluster 17. Consistent with the first pathway bundle, the dominant sources of 17 are paths 10 - 13 - 17, or 10 - 13 - 7 – 17, indicating 7 as a potential intermediate step before 17.

## Discussion

This study showed that unsupervised machine learning can identify predictive CT patterns associated with radiological disease progression in IPF. These patterns predict future outcome of patients. Identifying frequently occurring visual patterns in a patient cohort established a set of marker candidates. Training a model to recognize the temporal sequence of scan pairs based on these features selected candidates associated with radiological disease progression. They form a radiological disease progression signature. Clustering patients based solely on the similarity of these signatures yielded clusters with markedly different outcomes, even though clustering was blinded for survival data. This result was replicable on an external validation cohort collected at a different center in a different country. Finally, exploratory analysis of transition pathways of lung imaging patterns revealed routes from healthy to diseased tissue that may serve as basis for hypotheses regarding the underlying disease mechanisms based on the imaging data.

Machine learning algorithms typically provide means to automatically detect and quantify known markers in images based on supervised learning. Related recent work has shown the feasibility of machine-aided detection, quantitative imaging analysis, and pattern recognition in IPF/ILD [27, 28]. Humphries et al demonstrated a data-driven textural analysis visual system. Another study [29] showed adaptive multiple features lung texture analysis software for HRCT analysis.

In this study, we addressed three questions: First, can we identify lung pattern types associated with radiological disease progression in addition to those known and named such as ground glass opacities? This is relevant, since known patterns have limited power in reliably diagnosing fibrosing lung diseases [30, 31]. Consequently, data-driven means to expand our disease marker vocabulary could contribute to improving the diagnostic and prognostic capability of imaging. Our results show that in IPF the expansion of the marker vocabulary with additional patterns reliably associated to radiological disease progression is feasible.

Secondly, do groups of patients with different radiological disease progression signatures also have different future outcome? We grouped patients solely based on the dynamics of their signatures and found that in the study cohort these groups do have significantly different outcome. Even though signature construction and grouping were blinded to outcome, patients with similar signatures observed in CT shared risk for future outcome. To investigate to what extent signatures and grouping is directly transferrable, a replication experiment on an external cohort was performed. While the patient populations did share radiological IPF diagnosis, the center, country, and scanner manufacturers were different. Furthermore, they differed to some extent in their frequency of UIP, lung function, and smoking history. Despite that, we observed a comparable trend between groups assigned to the clusters identified in the study cohort but did not reach significance (*p* = .473). This suggests that dynamics carry predictive information, but while the newly identified signatures are transferrable, training on multicenter data with a more diverse disease composition may have benefits.

Finally, can we visualize shared the transition pathways of components, corresponding to frequently occurring changes of lung tissue during radiological disease progression. This might enable translating imaging features selected by machine learning models to biologically meaningful hypotheses regarding the underlying radiological disease process. The analysis demonstrates that these transition networks can be extracted from a patient population with follow-up examinations.

We evaluated if algorithms or human experts can identify radiological disease progression from imaging data, by letting them sort the temporal sequence of two consecutive CT scans. The algorithm and expert readers had overall comparable accuracy, with the algorithm (83%) in-between two readers (67% and 82%). Sorting mistakes can be due to either poor model performance, or lack of radiological disease progression. The overlap of errors between the readers and the algorithm (67% and 29%) suggests that at least in part lack of discernable radiological disease progression may lead to random sorting. Despite this, selecting features based on their capability of temporal sorting is a viable approach to mine imaging data for radiological disease progression markers.

Due to the high inter-observer variability in classifying IPF [32] and the varying effectiveness of treatments for IPF [33], it is important to identify novel radiological disease progression markers for diagnosis and monitoring of radiological disease progression and treatment in IPF. While prior work focused on quantifying the ratio of healthy to pathological lung tissue to obtain markers of radiological disease progression [34], we identified several patterns that exhibit consistent change behavior during radiological disease progression with unsupervised learning. The supervised prediction target of sorting cases serves as a proxy to identify novel predictor patterns exhibiting consistent change during radiological disease progression. Patterns are not used on their own, but as a multivariate signature, exploiting relationships between patterns. Post hoc qualitative analysis of the clusters in the progression signature revealed expected findings such as ground glass opacities, or overall lung density, but also regions surrounding small vessels. This is significant as vessel-related structures have also been reported to be an independent predictor of radiological disease progression in IPF, chronic hypersensitivity pneumonia, and unclassifiable fibrosis [35].

Location tracking during radiological disease progression enables the observation of how lung tissue transitions from one pattern to another over time. This augments marker patterns with transition pathways that may be a key to understanding the underlying pathomechanisms, and their signature visible in imaging.

Although our results of the machine learning methods show comparable performance in comparison with the expert radiologists, this work has several limitations. Our cohort is small in comparison with other machine learning results. Additionally, the gold standard for IPF diagnosis was based on imaging, possibly introducing bias into the model. The replication cohort contained imaging data with and without contrast enhancement, introducing heterogeneity into the imaging data. Despite this, the differences between the two clusters remained, although the HR was not significant.

In conclusion, unsupervised learning, together with a proxy task such as sorting the temporal sequence of examinations, can identify a stable set of radiological disease progression signatures in lung CT of IPF patients. Progression signatures identify groups in patients with different outcomes. Tracking the change of their components over time reveals transition pathways that may serve as a basis for further research.

## Abbreviations

ANTs: Advanced Normalization Tools
CT: Computed tomography
E1: Expert 1
E2: Expert 2
HR: Hazard ratio
IIP: Idiopathic interstitial pneumonias
ILDs: Interstitial lung diseases
IPF: Idiopathic pulmonary fibrosis
ML: Machine learning model
RF: Random forest
UIP: Usual interstitial pneumonia

## Declarations

### Guarantor

The scientific guarantor of this publication is Assoc. Prof. Helmut Prosch.

### Statistics and biometry

One of the authors has significant statistical expertise, Dr. Michael Weber.

### Methodology

• retrospective

• not applicable

• multicenter study
